# Reducing Extracellular Ca^2+^ Induces Adenosine Release via Equilibrative Nucleoside Transporters to Provide Negative Feedback Control of Activity in the Hippocampus

**DOI:** 10.3389/fncir.2017.00075

**Published:** 2017-10-10

**Authors:** Rebecca Diez, Magnus J. E. Richardson, Mark J. Wall

**Affiliations:** ^1^School of Life Sciences, University of Warwick, Coventry, United Kingdom; ^2^Department of Mathematics, University of Warwick, Coventry, United Kingdom

**Keywords:** adenosine, hippocampus, Ca^2+^-free activity, biosensors, equilibrative nucleoside transporters

## Abstract

Neural circuit activity increases the release of the purine neuromodulator adenosine into the extracellular space leading to A_1_ receptor activation and negative feedback via membrane hyperpolarization and inhibition of transmitter release. Adenosine can be released by a number of different mechanisms that include Ca^2+^ dependent processes such as the exocytosis of ATP. During sustained pathological network activity, ischemia and hypoxia the extracellular concentration of calcium ions (Ca^2+^) markedly falls, inhibiting exocytosis and potentially reducing adenosine release. However it has been observed that reducing extracellular Ca^2+^ can induce paradoxical neural activity and can also increase adenosine release. Here we have investigated adenosine signaling and release mechanisms that occur when extracellular Ca^2+^ is removed. Using electrophysiology and microelectrode biosensor measurements we have found that adenosine is directly released into the extracellular space by the removal of extracellular Ca^2+^ and controls the induced neural activity via A_1_ receptor-mediated membrane potential hyperpolarization. Following Ca^2+^ removal, adenosine is released via equilibrative nucleoside transporters (ENTs), which when blocked leads to hyper-excitation. We propose that sustained action potential firing following Ca^2+^ removal leads to hydrolysis of ATP and a build-up of intracellular adenosine which then effluxes into the extracellular space via ENTs.

## Introduction

The purine adenosine is a potent neuromodulator involved in many physiological and pathological processes (reviewed in Dunwiddie and Masino, 2001; Sebastião and Ribeiro, 2009; Borea et al., 2016). Adenosine acts via multiple subtypes of cell-surface G-protein-coupled receptors, with the high-affinity inhibitory A_1_ receptor the most widely expressed (reviewed in Fredholm et al., 2000). In the hippocampus activation of A_1_ receptors inhibits glutamatergic synaptic transmission and hyperpolarizes the membrane potential of pyramidal cells (Dunwiddie and Fredholm, 1989; Thompson et al., 1992). Adenosine is released during epileptiform activity both *in vivo* (measured with microdialysis) and *in vitro* (induced by removal of Mg^2+^; measured with biosensors) where it provides negative feedback to terminate bursts and delay the next burst of activity (During and Spencer, 1992; Dale and Frenguelli, 2009; Boison, 2015; Wall and Richardson, 2015). The activity-dependent release of adenosine into the extracellular space can occur through multiple mechanisms (reviewed in Wall and Dale, 2008) which include direct release of adenosine via equilbrative nucleoside transporters (ENTs; Lovatt et al., 2012; Wall and Dale, 2013) and indirect release as ATP by exocytosis from neurons (Pankratov et al., 2007) or glial cells (Newman, 2004; Pascual et al., 2005; Wall and Dale, 2013) to be metabolized to adenosine in the extracellular space. There is also evidence that adenosine can be released directly by exocytosis in the cerebellum (Klyuch et al., 2012). In the hippocampus, it appears that activity-dependent adenosine release occurs via a combination of different mechanisms which include exocytosis of ATP and transporter-mediated release (Wall and Dale, 2013).

During sustained pathological neural activity (Heinemann et al., 1977, 1981; Somjen and Giacchino, 1985), ischemia (Hansen and Zeuthen, 1981) and hypoxia (Silver and Erecińska, 1990) the extracellular concentration of calcium ions (Ca^2+^) is dramatically reduced. It is unclear what happens to adenosine signaling under these conditions, but since some of the adenosine release mechanisms are Ca^2+^ dependent, it would be predicted that adenosine release would also fall. However, it has been observed that removing extracellular Ca^2+^ actually enhances the amount of adenosine released during ischemia (Pedata et al., 1993; Frenguelli et al., 2007) and hypoxia (Dale et al., 2000) in the hippocampus, measured with HPLC and with biosensors. The mechanism for these increases in adenosine release are unclear but they maybe a consequence of the production of a paradoxical form of neural activity induced by Ca^2+^ removal. This neural activity can be observed in the hippocampus both *in vitro* (Haas and Jefferys, 1984; Konnerth et al., 1986; Agopyan and Avoli, 1988; Bikson et al., 1999) and *in vivo* (Feng, 2003). The mechanisms that increase neural excitability and synchrony are not fully understood but have been attributed to a number of processes including: reduced surface charge screening at the cell membrane (Frankenhaeuser and Hodgkin, 1957), inhibition of Ca^2+^-dependent K^+^ channels (Lancaster and Nicoll, 1987); reduced synaptic activation of GABAergic inhibitory interneurons (Bikson et al., 1999); electrical coupling via gap junctions (Perez-Velazquez et al., 1994) and extracellular electric-field effects (ephaptic transmission, Zhang et al., 2014). Removal of extracellular Ca^2+^ also enhances the opening of ion channels such as voltage-gated Na^+^ channels (Armstrong and Cota, 1999) and produces transient increases in extracellular K^+^ concentration that can facilitate the propagation of field bursts (Bikson et al., 2002). Here we have used biosensors and electrophysiology, to directly define what happens to adenosine signaling when extracellular Ca^2+^ is removed and to determine whether adenosine signaling plays a role in controlling the neural activity induced by Ca^2+^ removal.

## Materials and Methods

### Preparation of Hippocampal Slices

Sagittal slices of hippocampus (300–400 μm) were prepared from male Sprague-Dawley rats, at postnatal days 18–30. In accordance with the U.K. Animals (Scientific Procedures) Act (1986) rats were killed by cervical dislocation and decapitated. Hippocampal slices were cut with a Microm HM 650V Microslicer in cold (2–4°C) high Mg^2+^, low Ca^2+^ aCSF, composed of (mM): 127 NaCl, 1.9 KCl, 8 MgCl_2_, 0.5 CaCl_2_, 1.2 KH_2_PO_4_, 26 NaHCO_3_, 10 D-glucose (pH 7.4 when bubbled with 95% O_2_ and 5% CO_2_). Slices were stored at 34°C for 1–6 h in standard aCSF (127 NaCl, 1.9 KCl, 1 MgCl_2_, 2 CaCl_2_, 1.2 KH_2_PO_4_, 26 NaHCO_3_, 10 D-glucose). This study was carried out in accordance with the recommendations of the U.K. Animals (Scientific Procedures) Act (1986). All experiments were approved by the local Animal Welfare and Ethics Board at the University of Warwick (AWERB).

### Extracellular and Biosensor Recording from Hippocampal Slices

A slice was transferred to the recording chamber, submerged in aCSF and perfused at 6 ml/min (32°C). For extracellular recording, an aCSF-filled microelectrode was placed on the surface of stratum pyramidale in CA1. Extracellular recordings were made using a differential model 3000 amplifier (AM systems, Sequim, WA, USA). Signals were filtered at 3 kHz and digitized on line (10 kHz) with a Micro CED (Mark 2) interface controlled by Spike software (version 6.1, Cambridge Electronic Design, Cambridge, UK). Standard cylindrical microelectrode biosensors (50 μm diameter, Sarrisa Biomedical, Coventry, UK) were inserted into the slice in CA1 (Wall and Dale, 2013). Biosensor signals were acquired at 1 kHz with a Micro CED (Mark 2) interface using Spike (version 6.1) software. Zero Ca^2+^ aCSF consisted of standard aCSF with no Ca^2+^, 1 mM Mg^2+^ and 1 mM EGTA. For the experiments where high K^+^ aCSF was puffed onto area CA1 in the hippocampus, the high K^+^ aCSF had the same composition as zero Ca^2+^ aCSF but the total K^+^ concentration was elevated to 10–25 mM with KCl.

### Biosensor Characteristics

Biosensors (Sarissa Biomedical Ltd., Coventry, UK) consist of enzymes trapped within a matrix around a Pt or Pt/Ir (90/10) wire (Llaudet et al., 2003). Biosensors are cylindrical, and have an exposed length of ~500 μm and diameter of ~50 μm. Four types of sensor were used: null sensors, possessing the matrix but no enzymes, to control for non-specific electro-active interferents; biosensors containing adenosine deaminase, nucleoside phosphorylase and xanthine oxidase (responsive to adenosine, inosine and hypoxanthine: ADO biosensors); biosensors containing nucleoside phosphorylase and xanthine oxidase (responsive to adenosine metabolites inosine and hypoxanthine: INO biosensors); and finally ATP biosensors which consist of the entrapped enzymes glycerol kinase and glycerol-3-phosphate oxidase (Llaudet et al., 2005). Glycerol (2 mM) was included in the aCSF for experiments measuring ATP release, as glycerol is required as a co-substrate for ATP detection (Llaudet et al., 2005).

Biosensors show a linear response to increasing concentrations of analyte and respond rapidly (<1 s, Llaudet et al., 2003, 2005; Wall and Richardson, 2015; see Frenguelli and Wall, 2016 for a review of biosensor measurements in models of epilepsy). The lower limit of detection for adenosine biosensors is ~30–60 nM which would give a current of ~5–10 pA. Biosensors were calibrated with known concentrations (10 μM) of adenosine, inosine and ATP. To test the integrity of the screening layer 5-HT (10 μM) was applied. In many experiments, the composition of purines detected by ADO biosensors was not fully defined. Since ADO biosensors have an equal sensitivity to adenosine, inosine and hypoxanthine (Llaudet et al., 2003; Wall et al., 2007) the total concentration of purines detected was referenced to the calibration to adenosine to give μM or nM of purines. None of the drugs used in this study (8-cyclopentyltheophylline (8-CPT), 6-[(4-nitrobenzylothiol]-9-b-D-ribofuranosylpurine (NBTI) and dipyridamole) had any effects on biosensor sensitivity or the baseline current.

### Deconvolution and Reconvolution of Purine Waveforms

The amplitude of closely spaced waveforms produced by the release of adenosine can be difficult to measure accurately as subsequent pulses sit on the decay of proceeding ones. Following (Richardson and Silberberg, 2008), closely space release-events were deconvolved as in Wall and Richardson (2015) by removing the long decay τ_o_ component. The resulting sharper, well-spaced events were then cropped and reconvolved to yield isolated waveforms from which the amplitude and rise time could be accurately measured. All analysis was done in the JULIA programming environment.

### Whole-Cell Patch Clamp Recording from Pyramidal Cells

A slice was transferred to the recording chamber and perfused at 3 ml min^−1^ with aCSF at 32 ± 0.5°C. Slices were visualized using IR-DIC optics with a SliceScope (Scientifica) and a CCD camera (Scientifica, Bedford, UK). Whole-cell current clamp recordings were made from single or pairs of pyramidal cells in area CA1 of the hippocampus using patch pipettes (5–10 MΩ) manufactured from thick walled glass (Harvard Apparatus, Edenbridge, UK) and containing (mM): potassium gluconate 135, NaCl 7, HEPES 10, EGTA 0.5, phosphocreatine 10, MgATP 2, NaGTP 0.3 and biocytin 1 mg ml^−1^ (290 mOsm, pH 7.2). Voltage recordings were obtained using an Axon Multiclamp 700B amplifier (Molecular Devices, USA) and digitized at 20 kHz. Data acquisition and analysis was performed using Pclamp 10 (Molecular Devices). If the series resistance exceeded 14 mΩ than recordings were aborted, the mean series resistance was 11.2 ± 0.2 mΩ (*n* = 30). The standard I-V relationship was obtained by injecting step currents starting between −400 pA and −300 pA, incrementing by 100 pA until a regular firing pattern was induced. A plot of step current against average voltage response (measured at steady state after the activation of I_H_) around the resting potential was used to measure the input resistance (gradient of fitted line; as in Kaufmann et al., 2016).

### Drugs and Substances

All drugs were made up as 10–100 mM stock solutions, stored frozen and then thawed and diluted with aCSF on the day of use. Adenosine, inosine, 5 HT, dipyridamole, NBTI and 8-CPT were purchased from Sigma (Dorset, UK). ATP was purchased from Roche (Indianapolis, IN, USA). All substances were dissolved in distilled water except NBTI which was dissolved in DMSO (final concentration of DMSO in aCSF was 0.05%, which had no effect on biosensor or tissue responses).

## Results

### Characterization of Hippocampal Activity Induced by Removal of Ca^2+^

**Figure 1:**
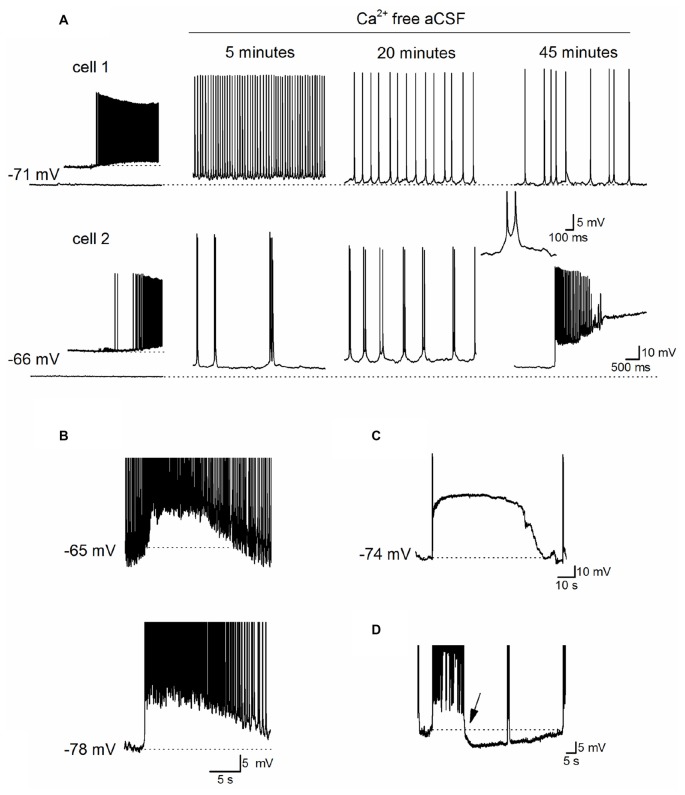
Characterization of Ca^2+^ free activity in the hippocampus. **(A)** Time-course of the effects of perfusing Ca^2+^-free aCSF (1 mM EGTA) on the activity recorded simultaneously from two pyramidal cells in area CA1 of the hippocampus. Inset: illustrates when Ca^2+^-free aCSF first began to induce neural activity in cell 1, and induces spontaneous events in cell 2 which maybe excitatory postsynaptic potentials (EPSPs). At 20 min, there are frequent short depolarizations with superimposed action potentials, a single example of such an event is illustrated in the inset (action potentials are truncated). After ~45 min there was a rapid depolarizing shift in the membrane potential in cell 2 that was not observed in cell 1. **(B)** Recordings from a pair of pyramidal cells where there was a simultaneous depolarization in both cells with a very similar time course. The top cell was active whereas the bottom cell was quiescent before the depolarization occurred. **(C)** Example of a large rapid depolarizing shift in membrane potential leading to depolarization block. The membrane potential remained depolarized (at around −16 mV) for ~70 s, repolarized and the pyramidal cell resumed firing. **(D)** Following a strong burst of activity, a prolonged hyperpolarization occurred (starting at arrow, duration ~50 s, dotted line is the membrane potential before the burst of activity). In **(D)** action potentials are truncated at −40 mV and this recording was not performed simultaneously with that in **(C)**.

Before assessing any adenosine release following the removal of extracellular Ca^2+^, we first characterized the neural activity produced when extracellular Ca^2+^ was removed. We investigated early changes in activity (0–1 h) following perfusion with zero-Ca^2+^ aCSF (zero Ca^2+^, 1 mM Mg^2+^ and 1 mM EGTA), unlike some previous studies where activity was measured 1–2 h after Ca^2+^ removal (Haas and Jefferys, 1984; Bikson et al., 1999). Whole-cell patch recordings were made from CA1 pyramidal cells in hippocampal slices (30 cells). This includes paired recordings (five pairs) so we could measure the homogeneity of activity across the network. Following perfusion with zero-Ca^2+^ aCSF pyramidal cells typically showed a pattern of initial depolarization (2–7 mV, mean membrane potential −58.5 ± 3 mV) with the firing of single action potentials at ~4–8 Hz (Figure 1A, cell 1 as previously reported in Konnerth et al., 1986). This was followed by a recovery of the membrane potential back to a value close to that observed at rest (after 5–10 min, mean resting potential −65 ± 2 mV), interrupted with short membrane potential depolarizations (~3–6 mV, mean depolarization 5.4 ± 0.5 mV occurring at a frequency between 2 Hz and 5 Hz) and superimposed bursts of action potentials (Figure 1A as previously described in Konnerth et al., 1986; Bikson et al., 2002). The precise profile of activity varied between recordings with some cells going straight into this phase, some cells remaining depolarized (Figure 1A, cell 2) and in some cells the membrane potential becoming hyperpolarized below that observed at rest with no action potential firing. Recordings from pairs of cells in the same field of view but not adjacent, showed that cells had individual patterns of activity (Figure 1A). This pattern of activity was often accompanied by occasional depolarizing shifts in the membrane potential (observed in 26 out of 30 recordings) which could be large enough to push the cell into depolarization block (Figure 1A, cell 2, Figures 1B–D). These depolarizations have been associated with waves of increased extracellular K^+^ observed with prolonged Ca^2+^ removal (over 1 h; Bikson et al., 2003) and could last for a few seconds to several minutes before the membrane potential hyperpolarized and the cell began firing again (Figure 1C). From paired recordings, it was found that these events could be localized (for example only observed in cell 2, Figure 1A) or could be synchronized (observed in both cells Figure 1B). In many recordings strong bursts of activity were followed by a prolonged membrane potential hyperpolarization (Figure 1D, mean duration 45 ± 10 s, *n* = 10). When the extracellular Ca^2+^ was washed back in, even after prolonged periods of activity (up to an hour), most pyramidal cells had resting membrane potentials (−71 ± 3 vs. −67 ± 7 mV) and IV relationships (steady state input resistance 121 ± 15 vs. 115 ± 13 mΩ) which were not significantly different to that observed at the start of the recording. Thus the removal of extracellular Ca^2+^ did not induce irreversible changes in pyramidal cell properties. Some recordings were lost before the return of extracellular Ca^2+^, data from these cells is included in our analysis.

### Endogenous Activation of A_1_ Receptors Controls Ca^2+^-Free Activity

**Figure 2:**
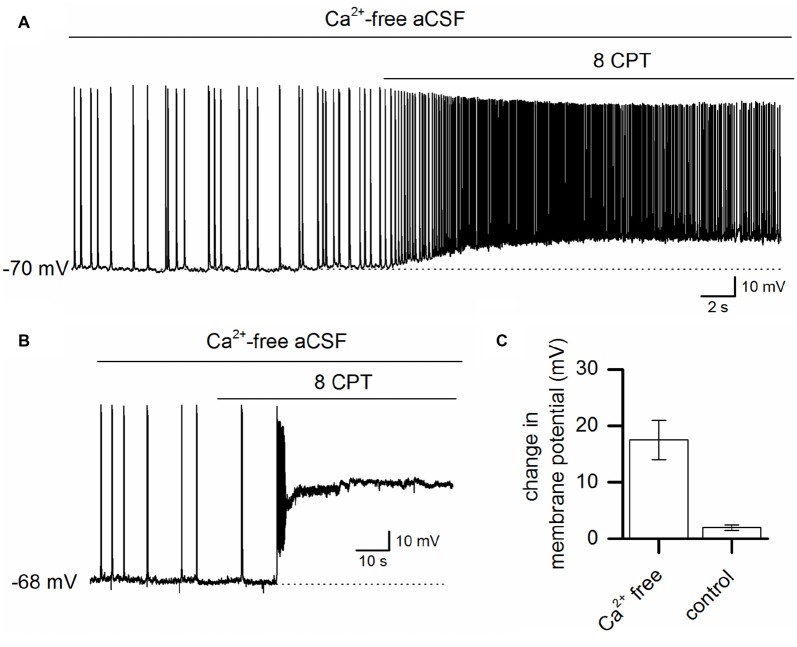
Ca^2+^ free-activity is suppressed by adenosine A_1_ receptor activation.** (A)** Whole-cell recording from a pyramidal neuron during Ca^2+^-free activity illustrating the effect of blocking adenosine A_1_ receptors with 8-cyclopentyltheophylline (8-CPT) (2 μM). **(B)** Example recording from a pyramidal cell where the application of 8CPT (2 μM) induced a large and rapid depolarizing shift in the membrane potential. The membrane potential remained depolarized for a long period (over 20 min). **(C)** The mean change (summarized for 8 cells, *P* < 0.001) in membrane potential produced by blocking A_1_ receptors with 8CPT in resting conditions (normal aCSF) and in Ca^2+^ free aCSF.

To assess whether endogenous activation of adenosine A_1_ receptors plays a role in controlling the activity induced by Ca^2+^ removal, the A_1_ receptor antagonist 8CPT (2 μM) was applied to slices. Application of 8CPT markedly depolarized the membrane potential (17.5 ± 3.5 mV, *n* = 8) and produced a significant increase in the action potential firing rate (Figure 2A). In some cells, this depolarization was followed by an irreversible depolarizing shift of the membrane potential and in other cells 8CPT immediately induced a depolarizing block (Figure 2B). Therefore endogenous activation of A_1_ receptors plays a key role in limiting Ca^2+^-free activity. In contrast, blocking A_1_ receptors in control aCSF had little or no effect on the resting potential of pyramidal cells (2 ± 0.5 mV, *n* = 5, Figure 2C) showing that when the network is quiescent there is little A_1_ receptor activation (in agreement with previous studies; Thompson et al., 1992). We therefore hypothesize that Ca^2+^-free-induced activity increases the extracellular concentration of adenosine leading to A_1_ receptor activation which feeds back to suppress activity.

### Extracellular Adenosine Concentration Is Increased in Ca^2+^ Free aCSF

**Figure 3:**
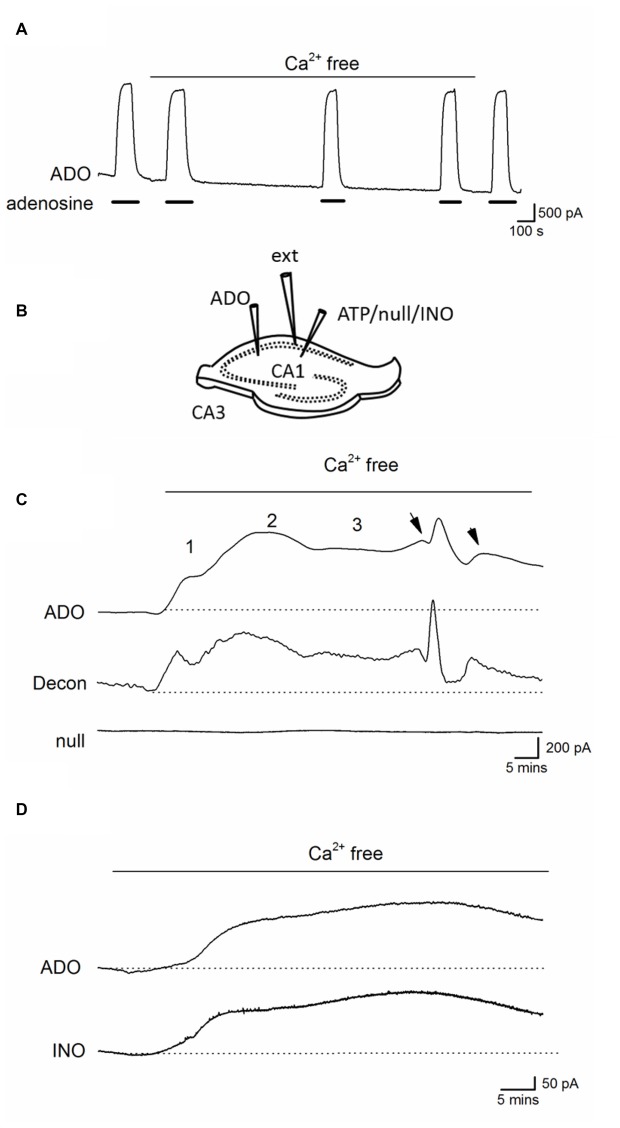
Ca^2+^-free activity increases the concentration of extracellular adenosine.** (A)** Trace from an ADO biosensor illustrating that removal of extracellular Ca^2+^ has no significant effect on the sensitivity to adenosine or baseline current (no slice was present). Adenosine (ADO) (10 μM) was applied during the filled bars. **(B)** Diagram showing positioning of electrodes and biosensors in a hippocampal slice. **(C)** Traces from an adenosine (ADO) biosensor and null sensor both placed in CA1 approximately 0.5 −1 mm apart. The ADO biosensor trace was deconvolved (Decon) with a time constant of 175 s to separate the release events. Application of Ca^2+^-free aCSF resulted in a large current on the ADO biosensor that was not observed on the null sensor. The current had three distinct phases with spontaneous events (arrows) superimposed on the sustained current in the latter phase. **(D)** Traces from ADO and INO biosensors during Ca^2+^ free activity. The traces are very similar and, given that the ADO biosensor measures both adenosine and inosine, suggests there is little or no adenosine component, which is consistent with adenosine metabolism before detection.

Before using adenosine (ADO) biosensors to directly measure any changes in the extracellular concentration of adenosine, we first tested whether changing the ionic environment had effects on the biosensor sensitivity to adenosine. Changing from standard aCSF to zero Ca^2+^ aCSF had no significant effect on the biosensor baseline or sensitivity to adenosine (Figure 3A) even with prolonged incubation (30 min). Thus biosensors can be used to accurately measure changes in extracellular adenosine induced by removal of Ca^2+^.

Biosensors were inserted into area CA1 of the hippocampus and tissue was allowed to recover (20–30 min, see Figure 3B for electrode arrangement). After application of Ca^2+^-free aCSF a large current was induced on ADO biosensors (peak value 741 ± 185 pA, Figures 3C,D *n* = 15) which was absent on null sensors and thus was due to the detection of purines (current equivalent to a concentration of 4.6 ± 1 μM of purines). This current had a number of distinct phases, which were particularly evident when the ADO biosensor traces were deconvolved and the phases were separated: there was an initial increase in baseline current (1, on Figure 3B), this reached an intermediate plateau (250 ± 50 pA, 1.8 ± 0.8 μM of purines) and then there was a second slower rising increase in purine concentration (2, on Figure 3B). This second component reached a peak, began to decline and then entered a long-lived third phase (3, on Figure 3B) that was often accompanied by superimposed spontaneous increases in purine concentration (arrows on Figure 3C). This third phase occurred at the same time that pyramidal cells exhibited a pattern of short membrane depolarizations and action potential firing. We used differential measurements with ADO and INO biosensors to determine whether adenosine or its metabolites were being measured (Figure 3D). The initial currents (phases 1 and 2) measured on ADO and INO biosensors were very similar in amplitude and kinetics, with no clear differential signal visible on the ADO biosensor, as would be expected if adenosine was directly detected (Figure 3D; Wall and Richardson, 2015).

### Spontaneous Purine Release Events Occur during Established Ca^2+^-Free Activity

**Figure 4:**
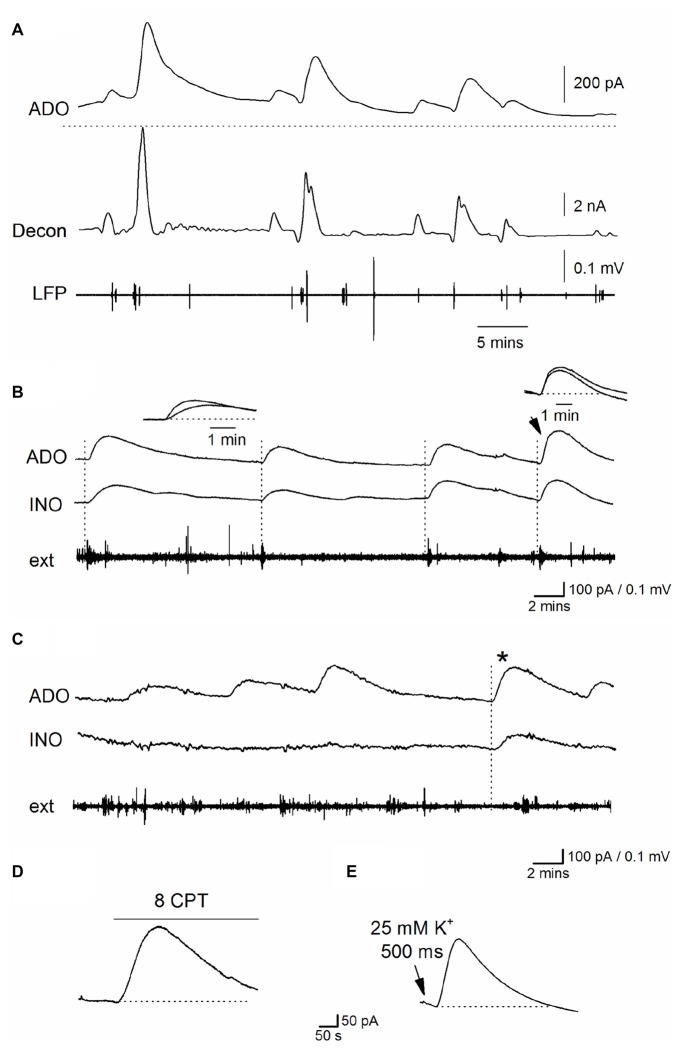
Properties of spontaneous adenosine waveforms during established Ca^2+^-free activity. **(A)** Traces from an ADO biosensor during established Ca^2+^ free activity. The top trace is the raw data showing spontaneous purine-release events, the dotted line shows the baseline current before activity began. The second trace (decon) shows the deconvolved trace where the slow component of decay (τ = 180 s) of the release events has been removed separating the overlapping pulses and highlighting the release dynamic’s. The bottom trace (LFP) is the LFP signal which has been extracted from the biosensor current trace. **(B)** Traces from ADO and INO biosensors and an extracellular electrode. In this example, spontaneous purine-release events were observed on both ADO and INO biosensors and were weakly correlated with bursts of activity on the extracellular electrode (dotted lines). **(C)** Traces from ADO and INO biosensors and an extracellular electrode. In this example the majority of the release events were observed only on the ADO biosensor and not the INO biosensor. Furthermore the events did not correlate with activity recorded by the extracellular electrode. The asterisk shows an event measured on both biosensors. Thus in this particular slice most of the events appear localized near where the ADO biosensor was inserted. **(D)** Application of 8CPT (2 μM) to block A_1_ receptors produced a current on the ADO biosensor. **(E)** Puffing (500 ms puff duration) of Ca^2+^ free aCSF with elevated K^+^ (25 mM) onto area CA1 induced a current on the ADO biosensor which mimicked spontaneous release events.

During established Ca^2+^-free activity (~30 min in Ca^2+^ free aCSF), spontaneous purine-release events were superimposed on a sustained elevated extracellular purine concentration in the majority of slices (9 out of 15, phase 3, Figures 4A–C). These waveforms occurred at a frequency of one every 4–10 min (mean interval between events 5.5 ± 4 min), amplitudes ranging between 50–400 pA (equivalent to 0.2–1.5 μM of purines), a 10%–90% rise-time of 51 ± 4 s and a slow, exponential decay (with a time constant ~200 s, Figure 4A). To investigate the spatial synchrony of these events across the slice, two biosensors were placed in CA1 (either two ADO biosensors or an ADO and INO biosensor), ~0.5 mm apart, with an extracellular electrode placed between them (as illustrated in Figure 3B). Figure 4B shows spontaneous purine-release events measured simultaneously (dotted lines) on both of the biosensors. It is known that adenosine is rapidly diluted by diffusion: its waveform is reduced to 1% of peak amplitude 100 μm away from the release source (Wall and Richardson, 2015). As the purine waveforms of similar amplitude are seen simultaneously at the two biosensors (separated by 0.5 mm) this must therefore reflect global release due to widespread, coordinated activity across CA1, rather than diffusive spread of purine from a single source. In contrast, in Figure 4C, the first three purine release events are only detected on one of the biosensors (ADO) and do not correlate with the activity on the extracellular electrode. It therefore appears that in this slice adenosine released is localized and occurs close to the ADO biosensor and this increase in concentration is dissipated over the distance between the biosensors. The final event (asterisk) is detected on both of the biosensors and thus activity at this time may be co-ordinated across the slice. This data suggests that purine release events can be localized or can spread globally across CA1 presumably co-ordinated by activity.

When release-events were detected on both ADO and INO biosensors, we compared the rise of the signals to see if we could separate components produced by adenosine and by its break-down product inosine. If adenosine is released into the extracellular space and then metabolized to inosine, a signal directly produced by adenosine would be expected to be faster than a signal produced by inosine, as it will be slowed by the intervening metabolism step. In this case the signal on the ADO biosensor would be faster than that on the INO biosensor. In contrast, if adenosine is metabolized to inosine before detection then the rise-time of events on ADO and INO biosensors will be similar as they are both detecting inosine. However, this is further complicated as the rise of biosensor signals also depends on the distance between the purine release site and the biosensor, with the rise slowed as the distance increases (Wall and Richardson, 2015). Across all biosensor recordings (*n* = 8) there was no consistent difference between the rise of the signals on ADO and INO biosensors (mean rise 52 ± 10 vs. 55 ± 8 s) suggesting adenosine is metabolized before detection. In Figure 4B, the first three release-events on the ADO biosensor have a faster rise than the signals on the INO biosensor, which is consistent with the detection of adenosine (as illustrated in the inset). However, the larger 4th event (arrow) has the same rise time on both biosensors (Figure 4B inset). This suggests that the difference in speed of rise between signals on the biosensors is likely to result from the different distances between the release sites and the biosensors rather than the direct detection of adenosine. These results suggest that adenosine is metabolized before detection as observed with other modes of neural activity and that rise is primarily determined by the distance between release sites and biosensor. The effects of blocking A_1_ receptors on activity (depolarization of membrane potential and increase firing rate, outlined earlier) confirms that adenosine is released (since inosine is inactive) and then metabolized before biosensor detection.

We hypothesize that these spontaneous increases in extracellular purine concentration are unlikely to arise from the frequent short depolarizations and associated action potential firing which is homogeneous and is therefore more likely to produce the tonic increase in adenosine concentration. Instead we propose they arise from the occasional periods of enhanced activity and rapid depolarization blocks that we observed with the whole cell patch clamp recordings. We have shown that periods of depolarization, with enhanced firing, can be localized or can spread across the tissue (Figure 1) which is consistent with the spatial dynamics of the biosensor signals (Figure 4). To test this further we carried out manipulations to increase activity and investigated whether this produces an increase in extracellular adenosine concentration as we predicted. Since blocking A_1_ receptors increases activity (Figure 2), this should also lead to an increase in extracellular adenosine concentration. We applied the A_1_ receptor antagonist 8CPT (2 μM) during established Ca^2+^ free activity and it reliably evoked a current on the ADO biosensor (290 ± 91 pA, equivalent to 1.6 ± 0.32 μM, Figure 4D, *n* = *5*). Since the periodic depolarizations that are observed are probably due to waves of extracellular K^+^ (Bikson et al., 2003), we predicted that puffing high K^+^ aCSF onto CA1 should mimic this effect. ACSF containing 10–25 mM K^+^ was puffed (500 ms) onto CA1 at least 30 min after removal of Ca^2+^. This reliably elicited large currents on the ADO biosensor (915 ± 350 pA, equivalent to 3.1 ± 0.93 μM, *n* = 7, Figure 4E) consistent with endogenous K^+^ waves releasing adenosine. Puffing zero Ca^2+^ aCSF with a standard concentration of K^+^ (3.1 mM) had no effect on the biosensor baseline current. This data is consistent with the spontaneous purine release events arising from periodic enhanced activity.

### Adenosine Release during Established Ca^2+^ Free Activity Is Blocked by ENT Inhibitors

**Figure 5:**
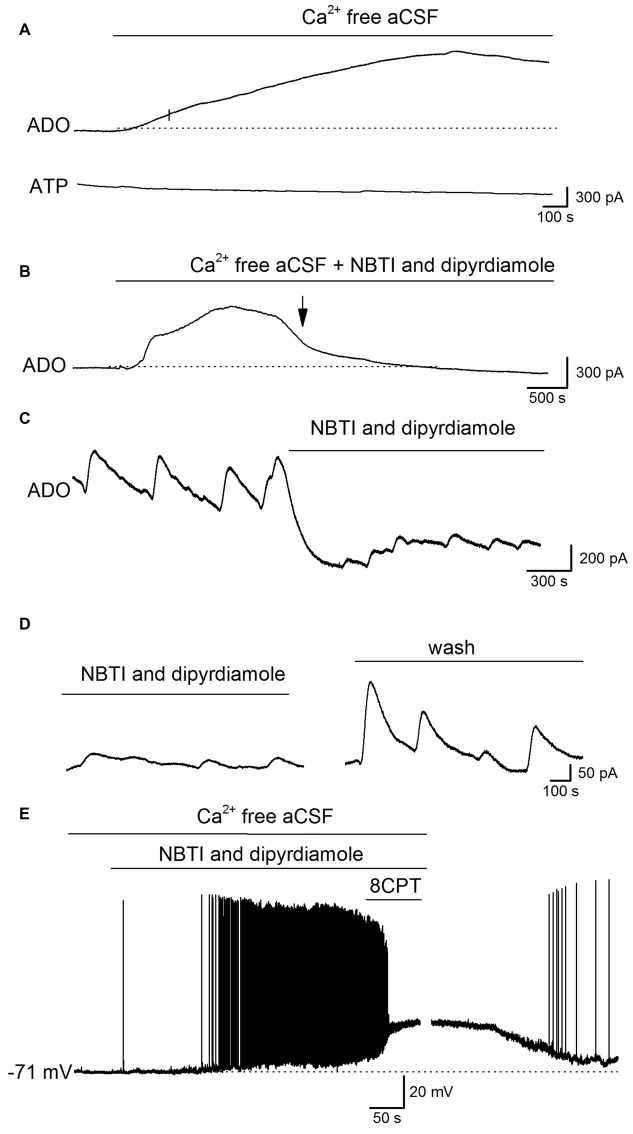
Adenosine is released via equilbrative nucleoside transporters (ENTs) during established Ca^2+^-free activity.** (A)** Traces from ADO and ATP biosensors during removal of extracellular Ca^2+^. Although there was a clear increase in the ADO biosensor current, no current was observed on the ATP biosensor. **(B)** Example trace from an adenosine biosensor (ADO). Application of Ca^2+^-free aCSF in the presence of the ENT blockers NBTI (5 μM) and dipyridamole (10 μM) induced the initial phases of the increase in extracellular adenosine (and metabolite) concentration but the late phase (phase 3) and the spontaneous release events were abolished (at arrow). **(C)** Example trace from an ADO biosensor. The spontaneous adenosine release events recorded after 30 min in Ca^2+^-free aCSF are greatly reduced in amplitude (~70%) and there is a fall in baseline current induced by the ENT inhibitors NBTI and dipyridamole. **(D)** Spontaneous release events recorded by an ADO biosensor increase in amplitude following wash of NBTI and dipyridamole. **(E)** Whole-cell patch-clamp recording from a CA1 pyramidal cell following 30 min incubation in Ca^2+^-free aCSF. Inhibition of ENTs (with NBTI and dipyridamole) induced depolarization and firing of action potentials. Application of 8CPT (2 μM) to block A_1_ receptors produced further depolarization and pushed the cell into depolarization block. Perfusing with aCSF containing Ca^2+^ led to a recovery of the membrane potential back to control values illustrating the viability of the recorded pyramidal cell.

What is the mechanism that links the increase in extracellular adenosine concentration with the activity induced by removing Ca^2+^? It could arise from either the release of ATP and its subsequent extracellular metabolism or the direct release of adenosine. The release of ATP by exocytosis would seem unlikely as there is no extracellular Ca^2+^ and no currents were observed on ATP biosensors (*n* = 6, Figure 5A). To investigate whether adenosine is directly released we used the ENT inhibitors NBTI (5 μM) and dipyridamole (10 μM, Wall and Dale, 2013). Experiments were carried out in two ways: first by pre-incubating slices with NBTI and dipyridamole (20–30 min) before removing the extracellular Ca^2+^ (NBTI and dipyridamole remained present throughout the experiment), and second by applying NBTI and dipyridamole after removal of extracellular Ca^2+^, during phase 3 (~30 min after Ca^2+^ removal). In slices pre-incubated with NBTI and dipyridamole the initial two phases of adenosine release persisted but the later sustained current and spontaneous events (phase 3) were either greatly reduced or abolished (mean inhibition 85 ± 10%, Figures 5B, *n* = 4). In a further six slices, NBTI and dipyridamole were applied after Ca^2+^ removal during phase 3 and they reduced the amplitude of both the sustained current (345 ± 40 to 120 ± 20 pA, mean inhibition 80 ± 8%) and the spontaneous release-events (reduced from 158 ± 56 to 46 ± 12 pA, mean inhibition 75.6 ± 5% Figure 5C). The effects of NBTI and dipyridamole were at least partially reversible in wash (Figure 5D, recovered to 72 ± 5% of control amplitude). This data suggests that adenosine is directly released via ENTs during established Ca^2+^-free activity.

### Blocking ENTs Increases Neural Activity

If the adenosine released via ENTs plays a negative feedback role to supress activity, then it follows that inhibition of ENTs should enhance neural activity. To test this, NBTI and dipyridamole were applied during whole-cell patch-clamp recordings from pyramidal cells in CA1 in Ca^2+^-free aCSF. Hippocampal slices were pre-incubated in Ca^2+^-free aCSF for at least 30 min before recordings were made, so that the early increases in extracellular adenosine concentration (not produced by ENTs) would have dissipated. Application of NBTI and dipyridamole depolarized CA1 pyramidal cells (mean depolarization 12 ± 3 mV, which is significantly (*P* < 0.001) greater than the amplitude of the short spontaneous baseline depolarizations 3.5 ± 0.5 mV) and increased their firing rate and some cells went into depolarization block (*n* = 6, Figure 5E). Application of 8CPT, to block A_1_ receptors in the presence of NBTI and dipyridamole further depolarized cells (Figure 5E) showing that some residual A_1_-receptor activation was still present. Thus the adenosine released via ENTs plays a major role in supressing activity, and when ENTs are inhibited activity is markedly enhanced.

## Discussion

We have shown that adenosine plays a key feedback role in controlling activity in the hippocampus induced by a reduction in extracellular Ca^2+^ concentration. A fall in extracellular Ca^2+^ can occur during strong epileptiform activity (Heinemann et al., 1986) and can initiate seizures *in vivo* (Feng, 2003) with hypocalcemia-induced seizures observed clinically (Han et al., 2015). Adenosine is released into the extracellular space during Ca^2+^-free activity to activate A_1_ receptors, leading to K^+^ channel activation and the hyperpolarization of pyramidal-cell membrane potential and reduction in pyramidal-cell firing rates. Without this endogenous A_1_-receptor activation, pyramidal cells become over-excited, fire action potentials at high rates and can move into long-lasting depolarization block. Once Ca^2+^-free activity is established, and Ca^2+^ dependent process, like exocytosis are blocked, the major mechanism for increasing the extracellular concentration of adenosine is the direct release of adenosine via ENTs. It would be interesting to test whether the additional adenosine released during ischemia (Pedata et al., 1993; Frenguelli et al., 2007) and hypoxia (Dale et al., 2000) induced by the removal of extracellular Ca^2+^ is also produced by ENTs.

### The Mechanism of the Initial Increase in Extracellular Adenosine Concentration

The mechanism for the increases in extracellular adenosine concentration, when slices are initially exposed to Ca^2+^ free aCSF, is unclear. These early phases of network activity are likely to be the result of multiple processes, as some residual extracellular Ca^2+^ will still remain, allowing the release of transmitter from active cells. A barrage of what appeared to be synaptic potentials was often observed at the start of Ca^2+^-free activity (Figure 1 inset). These initial phases of increased extracellular adenosine do not appear to arise from ATP release, as there was no clear signal measured on ATP biosensors; however, the rapid metabolism of ATP in the extracellular space, preventing the direct detection of ATP cannot be excluded. For example in Klyuch et al. (2012) and Wall and Dale (2013) ~50% of the adenosine release evoked by electrical stimulation arose from ATP release, as shown both pharmacologically and with transgenic mice, and yet in these studies no ATP could be directly detected using ATP biosensors. It also appears that the initial increases in extracellular adenosine concentration are not mediated via ENTs, as they persisted in slices which have been pre-incubated with the ENT inhibitors NBTI and dipyridamole.

We have shown that the adenosine released via ENTs plays a feedback role to control network activity during established Ca^2+^ free activity (20–30 min Ca^2+^ free). Thus inhibition of ENTs enhances the depolarization of CA1 pyramidal cells, increases their firing rate and pushes some cells into depolarization block. This is the opposite of what occurs under quiescent conditions when extracellular Ca^2+^ is present. Here the block of ENTs in the hippocampus leads to the accumulation of extracellular adenosine, activation of A_1_ receptors and the inhibition of transmitter release (Dunwiddie and Diao, 1994; Frenguelli et al., 2007). This illustrates the equilibrative nature of the transporters which can move adenosine into cells or release it into the extracellular space depending on the concentration gradient. Interestingly, the application of 8CPT, to block A_1_ receptors in the presence of NBTI and dipyridamole further depolarized cells (Figure 4E) showing that some residual A_1_ receptor activation is still present. This may result from incomplete block of ENTs or other mechanisms may also contribute to adenosine release. However it is clear that adenosine release via ENTs plays a major role in controlling Ca^2+^ free activity.

### Link between Activity and Adenosine Release

What mechanism links activity in Ca^2+^-free conditions to the release of adenosine? The simplest explanation is that prolonged neuronal depolarization and continual action potential firing results in the hydrolysis of large amounts of intracellular ATP, mainly by ion exchange pumps trying to maintain the membrane potential and extrude Na^+^ ions. It has been suggested that up to 55% of brain ATP consumption is used to pump Na^+^ ions out of cells under physiological conditions (Engel and Attwell, 2015). Presumably this proportion would be markedly increased during pathological activity (Engel and Attwell, 2015). Rapid production of significant amounts of adenosine inside neurons is likely to directly lead to adenosine efflux down the concentration gradient via ENTs because neurons do not express adenosine kinase (Studer et al., 2006) to convert the adenosine back into AMP. This is supported by the observations by Sims and Dale (2014) who reported that adenosine release, in response to AMPA receptor activation, is dependent on the activity of the Na^+^/K^+^ exchange pump. A reduction in extracellular Na^+^ concentration or application of ouabain, an Na^+^/K^+^ exchange pump inhibitor, markedly reduced evoked-adenosine release. In glial cells, large amounts of adenosine could saturate intracellular adenosine kinase, as it has a relatively low Km (~1–2 μM; Phillips and Newsholme, 1979). This would reduce or reverse the adenosine concentration gradient, leading to adenosine efflux via ENTs (Brundege and Dunwiddie, 1996).

Once Ca^2+^-free activity was fully established (~30 min in Ca^2+^ free aCSF) there were periodic spontaneous adenosine release events which were superimposed on the sustained extracellular concentration of adenosine (and its metabolites). Our data suggest that these spontaneous release events arise following strong activity probably induced by extracellular K^+^ waves : (1) in some slices these release-events were localized and only measured on one biosensor and in other slices the events were measured on both biosensors. This is consistent with the activity measured from whole-cell patch clamp recordings, where large depolarizations and depolarization blocks, could be either localized or could be synchronized across cells; (2) Bikson et al. (2003) showed, using ion sensitive microelectrodes that these depolarizations were produced by an increase in extracellular K^+^. These events could be mimicked by puffing high K^+^ onto CA1 (in Ca^2+^ free conditions) and this produced robust adenosine release; (3) application of 8CPT, to block A_1_ receptors, enhanced firing and also produced robust adenosine release and finally; (4) long lasting membrane potential hyperpolarizations often followed strong bursts of activity (see Figure 1D for example) or depolarization blocks, which could be produced by the released adenosine acting on A_1_ receptors. The biosensor waveforms are much longer (decay ~200 s) than the decay of the hyperpolarization (decay ~45 s) but this inconsistently can be explained by the fact that biosensors detect both adenosine and its metabolites, with the slow decay the result of diffusion of purines out of the slice (Wall and Richardson, 2015). An alternative explanation for the hyperpolarizations is the activation of Na^+^ or ATP dependent K^+^ channels (but not Ca^2+^-dependent K^+^ channels). This could potentially be tested by applying a cocktail of K^+^ channel blockers, since blocking of A_1_ receptors leads to marked depolarization, increased firing and in many cells depolarization block. These bursts of increased neural activity probably represent escape from adenosine suppression control. These may occur because the waves of increased extracellular K^+^ concentration weaken the hyperpolarizing effect of GIRK activation (as the outward K^+^ gradient will be reduced or reversed).

## Author Contributions

Experiments were designed by MJW and MJER. Experiments were carried out by MJW and RD. Data was analyzed by MJW and MJER. Article was written by MJW and MJER.

## Conflict of Interest Statement

The authors declare that the research was conducted in the absence of any commercial or financial relationships that could be construed as a potential conflict of interest.
